# Proportion of *Streptococcus agalactiae* vertical transmission and associated risk factors among Ethiopian mother-newborn dyads, Northwest Ethiopia

**DOI:** 10.1038/s41598-020-60447-y

**Published:** 2020-02-26

**Authors:** Mucheye Gizachew, Moges Tiruneh, Feleke Moges, Mulat Adefris, Zemene Tigabu, Belay Tessema

**Affiliations:** 1 Department of Medical Microbiology, School of Biomedical and Laboratory Sciences, College of Medicine and Health Sciences, University of Gondar, P. O. Box 196 Gondar, Ethiopia; 2 Department of Gynecology and Obstetrics, School of Medicine, College of Medicine and Health Sciences, University of Gondar, P. O. Box 196 Gondar, Ethiopia; 3 Department of Pediatrics, School of Medicine, College of Medicine and Health Sciences, University of Gondar, P. O. Box 196 Gondar, Ethiopia

**Keywords:** Microbiology, Diseases

## Abstract

Group B *Streptococcus* (GBS) vertical transmission causes fetal and neonatal colonization and diseases. However, there is scarcity of data in low-income countries including Ethiopia. We conducted a cross-sectional study on 98 GBS positive mothers, and their newborns to find proportion of vertical transmission. GBS was identified from swabs by using recommended methods and vertical transmission at birth was confirmed by the culture of body surface swabs of newborns within 30 minutes following birth. GBS positivity among swabbed specimens collected for other purposes was 160/1540 (10.4%); 98 were from 385 recto-vaginal swabs of pregnant women, and 62 were from 1,155 swabs of the 385 births. Of the 98 GBS positive cases, 62 newborns were GBS colonized with vertical transmission proportion of 63.3%(95% CI: 54.1–72.4%). We identified that the proportion of vertical transmission in this study was within the range of other many global studies, but higher than recently published data in Ethiopia. Maternal educational level, employment and lower ANC visit were significantly associated risk factors to GBS vertical transmission. Efforts need to be made to screen pregnant women during antenatal care and to provide IAP to GBS positive cases to reduce mother to newborn vertical transmission.

## Introduction

The achievement of the Sustainable Development Goal (SDG) 3 is a public health success to Ethiopia. However, progress in reducing under five and neonatal mortality rates remain slow. For instance, a 2016 Ethiopian Demographic and Health Survey (EDHS) report showed that under-5 mortality rate was 67 deaths per 1,000 live births, infant mortality rate, 48 deaths per 1,000 live births and neonatal mortality rate, 29 deaths per 1,000 births^1^. *Streptococcus agalactiae* (GBS), a gram positive cocci, is a leading cause of neonatal infections and deaths in developed countries and low- and middle-income countries, especially in Africa^2–4^.

Infant diseases caused by GBS is commonly categorized as early onset disease (EOD) which occurs in the first week of life (0–6 days), and late onset disease (LOD) that occurs from the 7^th^ days through 89^th^ days^5^. Vertical transmission of GBS can occur in pregnancy or during birth process from colonized pregnant women^6^. GBS that colonizes recttovagina of pregnant women can ascend and causes chorioamniotis, leading to fetal (in utero) and neonatal colonization^7^.

Western countries which implemented universal GBS screening of pregnant women, identification of associated risk factors, and offering Intrapartum antibiotic prophylaxis (IAP) at delivery were able to notably reduce the disease^8,9^. Despite the high rate of vertical transmission, most of the sub-Saharan African countries do not have clear strategies for prevention^9–11^. It could be due to limited data about GBS vertical transmission. Mother to newborn GBS transmission and its associated risk factors in Ethiopia, particularly, in the study area is scarce though one published report from the Eastern part of Ethiopia showed 45.02%.rate of vertical transmission^12^.

Though IAP lowers the risk of neonatal colonization and subsequent neonatal infections in the Western countries, culture-based screening of pregnant women at >35 weeks of gestation and provision of IAP to positive cases is not practiced in Ethiopia. The value of understanding the proportion of vertical transmission from pregnant women to their newborns right following birth is crucial to devise control and preventive strategies. In addition, to advance knowledge about the preventive strategies of vertically transmitting colonizing GBS, it is definitely worth making the effort to investigate the risk factors associated to vertical transmission in Ethiopia. This study, therefore, aimed to determine the proportion of vertical transmission of colonizing GBS and its associated risk factors among GBS positive pregnant women and their newborns in Northwest Ethiopia.

## Results

A total of 1,540 swab specimens from the 770 study participants were collected. Of these, 385 were the recto-vaginal swabs from the pregnant women (with >35 weeks of gestation), and the remaining 1,155 swabs were from the three body sites (ear, nasal and umbilicus) of the 385 newborns. The overall GBS positive rate among all the swabbed specimens was 160/1540 (10.4%). Out of the positive cases, 98 GBS-colonized cases were from 385 recto-vaginal swabs of pregnant women (Table 1). As shown in Table 2, the remaining 62/385 (16.1%) of the total newborns tested in this study were GBS colonized and of the total swabs processed, 62/1,155 (5.4%) were positive for GBS. Among the three newborn body surface sites swabbed, 19 (4.9%), 14 (3.6%), and 15 (3.9%) were only from ear, nasal and umbilical swabs of the 385 newborns (Table 2). The 14 newborns had GBS colonization on more than one body sites in which 7 (1.8%) were from nasal and umbilicus swabs, 2 (0.5%) from ear and umbilicus and 5 (1.3%) were from the nasal, ear and umbilicus swabs (Table 2).

**Table 1:** Characteristics of the study participants by vertical transmission at birth at University of Gondar Comprehensive Specialized Hospital, Northwest Ethiopia, 2019 (N = 98).

Characteristics		GBS test results of newborn at delivery		
Variables	Categories	Total, n (%)	Positive, n (%)	Negative, n (%)
Maternal age (year)	<25	73 (74.5)	48 (65.8)	25 (34.2)
	>25	25 (25.5)	14 (56.0)	11 (44.0)
Residence	Urban	77 (78.6)	51 (66.2)	26 (33.8)
	Rural	21 (21.4)	11 (52.4)	10 (47.6)
Education	Non-formal	30 (30.6)	15 (50.0)	15 (50.0)
	Primary	21 (21.4)	13 (61.9)	8 (38.1)
	Secondary	40 (40.8)	28 (70.0)	12 (30.0)
	Tertiary	7 (7.1)	6 (85.7)	1 (14.3)
Occupation	House wife	72 (73.5)	46 (63.9)	26 (36.1)
	Employed	21 (21.4)	13 (61.9)	8 (38.1)
	Others*	5 (5.1)	3 (60.0)	2 (40.0)
Gestational age (week)	<37	1 (1.0)	1 (100.0)	0 (0.0)
	>37	97 (99.0)	61 (62.9)	36 (37.1
History of still birth	No	94 (95.9)	59 (62.8)	35 (37.2)
	Yes	4 (4.1)	3 (75.0)	1 (25.0)
History of abortion	No	92 (93.9)	58 (63.0)	34 (37.0)
	Yes	6 (6.1)	4 (66.7)	2 (33.3)
History of neonatal death	No	93 (94.9)	60 (64.5)	33 (35.5)
	Yes	5 (5.1)	2 (40.0)	3 (60.0)
Gravidity	Primigravida	42 (42.9)	28 (66.7)	14 (33.3)
	Multigravida	56 (57.1)	34 (60.7)	22 (38.3)
Antenatal care (ANC) visit	0–3	67 (68.4)	46 (68.7)	21 (31.3)
	4–5	31 (31.6	16 (51.6)	15 (48.4)
Contraceptive use	No	12 (12.2)	7 (58.3)	5 (41.7)
	Yes	86 (87.8)	55 (64.0)	31 (36.0)
Meconium stained Amniotic fluid	No	92 (93.9)	58 (63.0)	34 (37.0)
	Yes	6 (6.1)	4 (66.7)	2 (33.3)
History of preterm delivery	No	95 (96.9)	60 (63.2)	35 (36.8)
	Yes	3 (3.1)	2 (66.7)	1 (33.3)
Premature rupture of Membrane (before labor onset)	<1 hr	74 (75.5)	48 (64.9)	26 (35.1)
	>1 hr	24 (24.5)	14 (58.3)	10 (41.7)
HIV status	No	92 (93.9)	59 (64.1)	33 (35.9)
	Yes	6 (6.1)	3 (50.0)	3 (50.0)
Duration of labor (hour)	4–12	82 (83.7)	54 (65.9)	28 (34.1)
	13–24	16 (16.3)	8 (50.0)	8 (50.0)
Sex of newborn	Male	56 (57.1)	35 (62.5)	21 (37.5)
	Female	42 (42.9)	27 (64.3)	15 (35.7)
APGAR^d^ Score at 1 minute	<7	8 (8.2)	5 (62.5)	3 (37.7)
	7–10	90 (91.8)	57 (63.3)	33 (36.7)
APGAR Score at 5 minutes	<7	2 (2.0)	2 (100.0)	0 (0.0)
	7–10	96 (98.0)	60 (62.5)	36 (37.5)
Newborn’s weight (Kg) median = 3.0	<2.5	11 (11.2)	6 (57.5)	5 (45.5)
	>2.5	87 (88.8)	56 (64.4)	31 (35.6)
**Resuscitation required**	No	91 (92.9)	58 (63.7)	33 (36.3)
	Yes	7 (7.1)	4 (51.1)	3 (42.9)
**Newborn to mother close contact**	No	18 (18.4)	12 (66.7)	6 (33.3)
	Yes	80 (81.6)	50 (62.5)	30 (37.5)

**Table 2:** Newborns GBS* colonization (n = 62) by their body surface sites at the University of Gondar Comprehensive Specialized Hospital, Northwest Ethiopia.

Newborn body site colonized	No. of GBS positive	Percentage (%)
Nasal swab only	19	4.9
Ear swab only	14	3.6
Umbilicus swab only	15	3.9
Nasal and ear swabs	0	0.0
Nasal and umbilicus swabs	7	1.8
Ear and umbilicus swabs	2	0.5
Nasal, ear and umbilicus	5	1.3
Total	62	16.1

### Demography, obstetric characteristics and maternal GBS colonization

In this study, 98 asymptomatically colonized pregnant women (≥35 gestational week of pregnancy) with GBS were analyzed to identify proportion of vertical transmission and its associated risk factors. As it is shown in Table 1, among the 98 GBS colonized mothers, 74.5% were below the age of 25 years old, 78.6% were urban dwellers, and 73.5% were house wives. Majority of the mothers had secondary education (40.8%) followed by those with non-formal education (30.6%; 57.1% were multigravida, and 68.4% had three or less times ANC follow up (Table 1).

### Demography and newborn GBS colonization

As shown in Table 1, among the total of 98 newborns participated in this study, 57.1% were males, 99.0% were delivered at >37 gestational weeks of pregnancy, 88.8% newborns were weighed 2.5 kg or more, 91.8% and 98.0% of the newborn had APGAR score of 7–10 at one and five minutes respectively. About 48 (65.8%), 51 (66.2%), 46 (63.9%) of the newborns colonized with GBS were born from colonized mothers with the age of <25 years old, urban dwellers, and housewives respectively. Those newborns with APGAR score of <7 at five minutes and those with weight of 2500 gram or more had more colonization rate (Table 1).

### Proportion of vertical transmission and its predictors

The proportion of vertical transmission of GBS from the pregnant women to the newborn was 62/98 (63.3%). To estimate the relative contribution of each factor to vertical transmission of GBS, adjusted odds ratio was calculated by using the multivariable logistic regression. This analysis showed that maternal educational status, maternal occupation, and maternal ANC follow up were significantly associated to vertical transmission

Newborns born to those mothers who had primary and secondary educational level were 32.7 times (AOR = 32.657; 95% CI: 2.271, 469.541), and 18.8 times (AOR = 18.849; 95% CI: 1.276, 278.483) more likely to have increased risk of being colonized respectively. This wide confidence interval might be owing to the sample size. Moreover, those newborns who were delivered from the employed mothers had about 5 times (AOR = 4.599; 95% CI: 1.096, 19.297) and those who were from pregnant women who had 4 to 5 times of ANC visit were less likely 0.21 time (AOR = 0.209; 95% CI: 0.063, 0.696) to transmit GBS to their neonate (Table 3). were less likely (0.21 AOR…) to transmit GBS to their neonate’.

**Table 3:** Results of multivariate logistic regression model on risk factors associated with vertical transmission of GBS at birth at University of Gondar Comprehensive Specialized Hospital, Northwest Ethiopia.

Characteristics		Total, n (%)	COR*; 95% CI	*p-value*	AOR**, 95% CI	*p-value*
Variables	Categories					
Maternal age (year)	<25	73	1	1		
	>25	25	1.4 (0.59, 3.81)	0.38		
Residence	Urban	77	1	1		
	Rural	21	1.7 (0–67, 4.74)	0.24		
Maternal education	Non-formal education	30	1	1	1	1
	Primary	21	6.0 (0.64, 56.06)			
	0.11	32.6 (2.27, 469.54)	0/01			
	Secondary	40	3.6 (3.73, 36.57)	0.26	18.8 (1.28, 278.48)	0.03
	Tertiary	7	2.5 (0.28, 23.73)	0.40		
Maternal occupation	House wife	72	1	1	1	1
	Employed	21	1.1 (0.399, 2.97)	0.87	4.5 (1.09, 19.29)	0.03
	Others*	5	1.2 (0.18, 7.52)	0.86	—	—
Gestational age (week)	<37	1	0.000 (0.000, -)	1.00		
	>37	97	1	1		
History of still birth	No	94	1	1		
	Yes	4	0.56 (0.06, 5.61)	0.62		
History of abortion	No	92	1	1		
	Yes	6	0.85 (0.15, 4.90)	0.86		
History of neonatal death	No	93	1	1		
	Yes	5	2.7 (0.43, 17.15)	0.28		
Gravidity	Primigravida	42	1.3 (0.56, 2.99)	0.55		
	Multigravida	56	1	1		
Antenatal care visit	0–3	67	1	1	1	1
	4–5	31	2.1 (0.86, 4.92)	0.12	0.2 (0.06, 0.69)	0.01
Contraceptive use	No	12	1.3 (0.37, 4.33)	0.706		
	Yes	86	1	1		
Meconium stained Amniotic fluid	No	92	1	1		
	Yes	6	0.85 (0.15, 4.90)	0.86		
History of preterm delivery	No	95	1	1		
	Yes	3	0.8 (0.08, 9.79)	0.90		
Premature ROM**	<1 hr	74	1	1		
	>1 hr	24	0.9 (0.07, 9.79)	0.90		
HIV status	No	92	1	1		
	Yes	6	1.7 (0.43, 9.37)	0.49		
Duration of labor (hour)	4–12	82	1	1		
	13–24	16	1.9 (0.65, 5.68)	0.23		
Sex of newborn	Male	56	1	1		
	Female	42	0–9 (0.40, 2.13)	0.85		
APGAR^**#**^ Score at 1 minute	<7	8	0–9 (0.22, 4.29)	0.96		
	7–10	90	1	1		
APGAR Score at 5 minutes	<7	2	0.000 (0.000, −)	0.999		
	7–10	96	1	1		
Newborn’s weight (Kg) median = 3.0	<2.5	11	0.66 (0.19, 2.35)	0.53		
	>2.5	87	1	1		
Resuscitation required	No	91	1	1		
	Yes	7	1.3 (0/28, 6.25)	0.73		
Newborn to mother close contact	No	18	0.76 (0.61, 0.94)	0.01		
	Yes	80	1	1		

^a^Crude odds ratio; ^b^Adjusted odds ratio; ^#^Appearance, Pulse, Grimace, Activity, and Respiration; *Student; **Rupture of membrane.

## Discussion

*Streptococcus agalactiae* has remained as an important cause of infection in the perinatal period. GBS is of particular interest because of the fact that the IAP given to colonized mothers can reduce the burden of early-onset neonatal diseases though it has limited impact on the late onset GBS associated neonatal diseases. This requires screening of pregnant women at the third trimester of pregnancy or in labor and administration of antibiotics to those colonized. The reported maternal carriage rate of GBS when multiple sites were cultured ranges from 10 to 41%, while vertical transmission varies from 40% to 70%^13,14^. This variation in the prevalence of colonization might be associated with geographic region, socio-demographic status, and, sexual activity. Reduction of this vertical transmission of GBS to the newborn has been a priority over the past three decades. The method that has proved the most successful has been screening of all pregnant women during pregnancy, and provision of intrapartum antibiotics to colonized women in labor. However, the GBS screening and IAP provision service is not practiced particularly in the study site and in Ethiopia at large. Using this strategy, GBS infection among newborn in the USA has been reduced from 1.7–1.9 per 1000 live births in the early 1990s, to 0.34–0.37 per 1000 newborn in 2008^15^.

We assumed that this was vertical transmission since the collection of specimens was made immediately after birth without any further handling and/or wiping of the newborns makes acquisition of GBS from other sources unlikely. The proportion of vertical transmission of GBS from pregnant mother to the newborn in the current study (63.3%.) is therefore within the ranges of global reports. However, when it is compared to the individual reports generated around the world, this proportion of vertical transmission is higher than studies conducted in USA (53.8%)^16^; China (7.6% to 16.7%)^17–19^; Bangladesh 38.0%^20^; Kuwait (35.5%)^13^; Eastern Ethiopia (45.02%)^21^; Central and Southern Ethiopia where the overall vertical transmission rate was 54.1% in which, 56.8% was recorded from the Adama Hospital Medical College; and 49.2% from Tikur Anbessa Specialized Hospital, and 59.1% from Hawassa Referral Hospital^22^. These discrepancies might be due to variations in demographic characteristics, geographic location, and service availability as described in a report from elsewhere in the world^7^. On the other hand, the proportion of vertical transmission recorded in the current study was comparable with other studies done in different parts of the world, which is in the range of 40–70%^13,14^, including recent reports from India (65.0%)^23^; USA (61.5%)^24^; and Ethiopia, Hawassa Referral Hospital (unpublished data) (59.1%)^22^. High rate of vertical transmission contributes to high neonatal and maternal morbidity and mortality due to GBS. Vertical transmission of GBS is preventable and thus, health care providers and policymakers need to consider this in their maternal and neonatal mortality reduction strategies^25^.

Factors which may determine vertical transmission of GBS from colonized mother to newborn is not clearly identified, mainly in low-income countries. Thus, investigating the risk factors which might be associated to vertical transmission would be useful to devise preventive strategies. The three risk factors that were significantly associated with vertical transmission of GBS from the asymptomatically colonized mother to newborn we found in the current study were maternal educational status; maternal occupation and ANC follow up. Those mothers who had primary and secondary educational level and those with employed mothers had 65.7%, 84.9% and 59.9% more likely to have a risk to transmit the colonizing GBS vertically to their newborns as compared to their counterparts’ respectively. The pregnant women who had 4–5 times of ANC visit during their current pregnancy had 20.9% less likely to transmit the colonizing GBS to their newborns vertically.

Various studies showed that maternal age, parity, marital status, education, occupation, and high body mass index might be associated with GBS colonization in pregnancy^26,27^ and this would cause for the vertical transmission to newborns. It is because colonization of the maternal birth canal with is assumed to be a major source of newborn colonization which in turn is an important risk factor for the morbidity and mortality of neonates with early-onset GBS disease.

## Conclusion

The current study revealed that the proportion of vertical transmission of GBS from asymptomatic mother to newborn was found to be within the ranges of reports of the world. Maternal primary and secondary school educational level, maternal employment, and 0 to 3 times of ANC visit during the current pregnancy were significant risk factors for GBS vertical transmission from colonized mothers to their newborns. More studies in different parts of the country are needed to establish guidelines for GBS screening and IAP use in Ethiopia. Prospective studies are also needed to evaluate the GBS disease burden in Ethiopian mothers and infants.

## Materials and Methods

### Ethical considerations

It was conducted after we secured the ethical approval by the Ethical Review Committees of the University of Gondar (IRB) (R.No. O/V/P/RCS/05/478/2015) as per the declaration and regulation of Helsinki as a statement of ethical principles. Permission was obtained from the Hospitals administrative bodies. The study participants were informed about the value of the study before we are gaining to collect any data or samples. Informed consent and/or assent were obtained from the study participants. The ear, nasal and umbilicus swabs were collected by the experienced midwives and processed at the bacteriology laboratory by using the CDC 2010 guideline of prevention of perinatal GBS disease^15^. Participants (mothers) had full right to continue or withdraw their newborns from the study. Confidentiality of all the participants’ information were maintained throughout the study

### Study area

The study was conducted at the University of Gondar Comprehensive Specialized Hospital in the Amhara National Regional State, Northwest Ethiopia. The reports of the Central Statistical Agency of Ethiopia (CSA) population projection and the Amhara National Regional State Health Bureau showed that the region has about 20,018,988 people and nearly half of it is the females. The University of Gondar Comprehensive Specialized Hospital is one of the oldest hospitals in Ethiopia, and has around 450 to 600 pregnant women admission services per month. The hospital has the service and teaching laboratory facilities where microbiological identification takes place^28,29^.

#### Study design and period

A hospital based prospective cross-sectional study was conducted from December 2016 to November 2017.

### Population

#### Source population

All pregnant women and their newborns that were attend at the University of Gondar Comprehensive Specialized Hospital in the Amhara National Regional State, Northwest Ethiopia.

#### Study population

Pregnant women with gestational age of pregnancy >35 weeks and who were colonized with GBS, and their newborns who were attended at the University of Gondar Comprehensive Specialized Hospital in the Amhara National Regional State, Northwest Ethiopia.

### Inclusion and exclusion criteria

#### Inclusion criteria

Pregnant women who were colonized with GBS at their labor with >35 weeks of gestational age of pregnancy, and their corresponding newborns delivered via vagina

#### Exclusion criteria

Pregnant women who were/had severely ill, mentally unstable, emergency room, current vaginal bleeding, current use of systemic antibiotics active to GBS within two weeks prior to data collection, and newborns delivered other than through birth canal.

### Study variables

#### Dependent variable

Proportion of GBS vertical transmission.

#### Independent variables

The socio-demographic factors such as age, education, address and occupation, and the obstetrics/ gynecology like the use of contraceptive methods, ANC follow up, gravidity, parity, breast feed (close contact with the mother), prolonged labor, Prolonged rupture of membrane, premature ROM, pre-term delivery, low birth weight; intrapartum fever ≥38 °C, history of foetal losses, history of neonatal deaths.

#### Sample size determination

Three hundred eight five pregnant women who were in active labor at their gestational age of 35 weeks or more and 385 newborns following delivery were investigated for their GBS colonization that was designed for a different study. In such a study, 98 women were found to be GBS postive and all these GBS positive pregnant women and their corresponding newborns (98) were included in this study to determine the proportion of vertical transission.

#### Data collection, sampling technique and laboratory procedures

The socio-demographic and biological data were collected from the pregnant women (with >35 gestational weeks of pregnancy) at the point of labor and their newborns by trained midwives and laboratory technologists from the maternity ward of the hospital until the prespecified sample size was reached.

### Data collection tools

#### Questionnaire

Socio-demographic data were collected from the study participants by using the semi-structured questionnaire to investigate risk factors associated to newborns GBS colonization (vertical transmission). Questionnaires were prepared by using the published studies tailored based on our objective. The questionnaire were first prepared in English and translated into Amharic, the language which the study participants speak. After the data were collected by using the Amharic language, the response of each questionnaire was re-translated into English for analysis and report.

#### Biological specimen collection

Rectovaginal swabs from the pregnant women, and ear, nasal and umbilicus swabs from the newborns were collected and analyzed by using the Centers for Diseases Control and Prevention (CDC), 2010 and the Clinical and Laboratory Standards Institute (CLSI), 2014 guidelines^15,30^ at the microbiology laboratory of the Department of Medical Microbiology, School of Biomedical and Laboratory Sciences, University of Gondar.

### Swab culture

The rectovaginal, ear, nasal, and umbilicus swabs collected were transported by using the Aims transport medium and placed into the Todd-Hewitt selective enrichment broth containing colistin (10 µg/ml) and naldixic acid (15 µg/mL) (Cart Roth GmbH + Co. KG-Schoemperlensrr. 3-5-D-76185 Karisruhe, Germany). It was incubated at 37 °C in 5% CO_2_ for 24 hours. The growth was sub-cultured onto 5% defibrinated sheep-blood agar and the isolates were identified by using the colony morphology, Gram staining reaction, β - hemolytic features, and CAMP test. The culture plates were re-incubated for another 24 hours and inspected again as the β - hemolytic colonies were not observed during the first 24 hour incubation time. The β - hemolytic colonies morphologically consistent with GBS were sub-cultured onto 5% defibrinated sheep blood agar and subjected to CAMP test for presumptively identification. The CAMP test was done by inoculating the known *Staphylococcus aureus* onto 5% defibrinated sheep blood agar down the center of the plate with a wire loop. GBS was streaked in a straight line perpendicular to the *S. aureus* within 2 mm far. The plate was then incubated at 35 °C for 24 hours. A positive CAMP result was indicated by an arrowhead-shaped enhanced zone of beta-hemolysis in the area between the GBS and *S. aureus* with the arrow-point towards the *S. aureus* streak.

#### Quality control

About 5% of the questionnaire and the protocol were pre-tested to check their suitability. Data cleaning was done daily, and *Streptococcus agalactiae* (ATCC 12386), *Enterococcus faecalis* (ATCC 29212); *Streptococcus pyogenes* (ATCC 19615), *Staphylococcus aureus* (ATCC 29213) and *Escherichia coli* (ATCC 25922) were used as quality control.

#### Data analysis

The excel spread sheet was used to enter the data, and was cleaned and exported to IBM SPSS version 20 (Chicago, IL, USA) and analyzed. Results were reported by using words, and tables. Descriptive statistics was used to summarize characteristics of the study participants. The association between the outcome variable (proportion of vertical transmission of GBS) and each independent variable (demography and clinical factors) was analyzed by using the multivariable logistic regression model. All the variables were entered into the multivariable logistic regression by using backward LR selection procedure to control the confounding effect and to retain only the statistically significant variables in the final model. Association between the outcome and the independent variables was calculated by using the adjusted odds ratio at a p-value < 0.05 and 95% confidence interval. Assumption of goodness of the model was checked by Hosmer-lemeshow test (p = 0.828).

## Data Availability

All data generated or analyzed during this study are included in this manuscript and when there is a need of raw data, it would be available through formal request of the corresponding author.
